# Differentially Expressed Genes in the Brain of Aging Mice With Cognitive Alteration and Depression- and Anxiety-Like Behaviors

**DOI:** 10.3389/fcell.2020.00814

**Published:** 2020-08-28

**Authors:** Mengqi Li, Songxue Su, Weihua Cai, Jing Cao, Xuerong Miao, Weidong Zang, Shichao Gao, Ying Xu, Jianjun Yang, Yuan-Xiang Tao, Yanqiu Ai

**Affiliations:** ^1^Department of Anesthesiology, Pain and Perioperative Medicine, The First Affiliated Hospital of Zhengzhou University, Zhengzhou, China; ^2^Neuroscience Research Institute, Zhengzhou University Academy of Medical Sciences, Zhengzhou, China; ^3^Department of Anatomy, College of Basic Medicine, Zhengzhou University, Zhengzhou, China; ^4^Department of Anesthesiology and Intensive Care, Third Affiliated Hospital of Second Military Medical University, Shanghai, China; ^5^Department of Pharmaceutical Sciences, School of Pharmacy and Pharmaceutical Sciences, The State University of New York, Buffalo, NY, United States; ^6^Department of Anesthesiology, New Jersey Medical School, Rutgers, The State University of New Jersey, Newark, NJ, United States

**Keywords:** cognitive dysfunction, depression, anxiety, RNA sequencing, aging mice

## Abstract

Despite the great increase in human lifespan with improved medical care, the physiological and pathological changes such as memory and cognitive disorders and associated anxiety and depression are major concern with aging. Molecular mechanisms underlying these changes are little known. The present study examined the differentially expressed genes (DEGs) and the genes with differentially expressed isoforms in three brain regions, anterior cingulate cortex (ACC), amygdala and hippocampus, throughout the lifespan of mice. Compared to 2-month old mice, both 12- and 24-month old mice displayed memory and cognitive impairments in the Morris water maze, Y-maze, and novel object recognition tests and depression- and anxiety-like behaviors in the tail suspension, forced swimming, open field, and elevated plus maze tests. RNA sequencing analysis identified 634 and 1078 DEGs in ACC, 453 and 1015 DEGs in the amygdala and 884 and 1054 DEGs in hippocampus in the 12- and 24-month old mice, respectively. Similarly, many genes with differentially expressed isoforms were also identified in these three brain regions in the 12- and 24-month old mice. Further functional analysis revealed that many DEGs and the genes with differentially expressed isoforms in the ACC and amygdala were mapped to depression- and anxiety-related genes, respectively and that a lot of DEGs and the genes with differentially expressed isoforms in hippocampus were mapped to cognitive dysfunction-related genes from both 12- and 24-month old mice. All of these mapped DEGs and the genes with differentially expressed isoforms were closely related to neuroinflammation. Our findings indicate that these neuroinflammation-related DEGs and the genes with differentially expressed isoforms are likely new targets in the management of memory/cognitive impairment and emotional disorders during the aging.

## Introduction

With rapid socio-economic development, the life expectancy of human being is increasing (Beard et al., 2016). However, age-related disorders such as Alzheimer’s disease (AD) and dementia afflict vast majority of aged patients and become most significant public health issues and considerable challenges around the world (Kanasi et al., 2016). Indeed, the aging process may involve a series of behavioral and psychological dysfunction due to neurodegenerative changes in cell homeostasis and biological pathways (Gurkar and Niedernhofer, 2015). Clinical investigation indicates that aged patients with dementia often present clusters of behavioral symptoms such as memory and cognitive disorder, depression, anxiety, and neuropathic pain (Frisoni et al., 1999; Herrup, 2010; Salthouse, 2012; Harada et al., 2013). Although cognitive and functional impairment as the hallmark of aging is often emphasized, neuropsychiatric symptoms are also directly responsible for the reduced quality of life and the increased rates of disability in patients and their families. Current treatments for these age-related disorders are unsatisfactory and/or produce adverse effects at least in part due to incomplete understanding of molecular mechanisms underlying these disorders. Therefore, identifying the differentially expressed genes (DEGs) in the cognitive impairment-, depression-, or anxiety-associated brain regions of aging mice is a key step for searching new targets for novel treatments and preventative tactics for the age-related disorders.

Several brain regions such as hippocampus, amygdala and anterior cingulated cortex (ACC) are involved in the aging process and participate in the pathological processes of age-related cognitive and emotional disorders (Killgore and Yurgelun-Todd, 2004; Etkin et al., 2006; Liu W. et al., 2017; Martin et al., 2017; Ewbank et al., 2018; Piel et al., 2018). Indeed, the gene and protein networks among these three brain regions (Killgore and Yurgelun-Todd, 2004; Etkin et al., 2006; Ewbank et al., 2018; Piel et al., 2018) directly regulate cognitive dysfunction and depression/anxiety-like behaviors (Chao et al., 2016; Liu W. et al., 2017; Martin et al., 2017; Scalici et al., 2017; Yan et al., 2018). Although there are numerous hypotheses for understanding the pathogenesis of age-related memory deficits and emotional dysfunction, the dysregulation of genes and proteins in brain regions during aging has been widely acknowledged (Zhang et al., 2016; Alam et al., 2018; Holmes et al., 2018; Mehta et al., 2018). Several studies have identified DEGs between young- and aged-mice, but most of them focused on only one brain region and/or at a one-aged time point (Reichwald et al., 2009; Cribbs et al., 2012). Thus, it is imperative to obtain more thorough molecular profiles with aging by examining different gene changes in multiple brain sites throughout multiple time points during the lifespan.

To better understand the network of gene changes in the ACC, hippocampus and amygdala with aging, the present study focused on gene expression that changed in these three brain regions throughout the lifespan of mice. To this end, we employed the 2-, 12-, and 24-month old mice and first observed memory and cognitive performance, depression- and anxiety-like behaviors and the responses to nociceptive stimulation. We then collected ACC, amygdala and hippocampus from these mice and performed next-generation RNA sequencing with a higher sequencing depth and without mRNA poly-A tail selection. Finally, we analyzed and compared the transcriptome profiles in these regions among these different age mice. Our findings may provide novel information that may be used to identify new targets for the prevention and treatment of age-related neurodegenerative disorders.

## Materials and Methods

### Animals

The 7-week old C57BL/6J wild-type male mice were obtained from Charles River Laboratory (Beijing, China) and Sino-British SIPPR/BK Lab (Shanghai, China) in this study. Animals were housed in a temperature-controlled room and exposed to a standard 12 h light-dark cycle and normal illumination environment. Three groups of different age mice (2-, 12-, and 24-month old; 8 mice/group) were used. The animals had at least 1 week for acclimation to facility environment before behavioral tests, which were carried out in a quiet room between 10:00 a.m. and 4:00 p.m. (daytime). The mice were acclimated to experimental apparatus for 45 min before the tests. All behavioral tests were done within 1 week. All procedures were conducted in accordance with the ethical guidelines of the National Institutes of Health and the International Association for the Study of Pain and approved by the Animal Care and Use Committee of Zhengzhou University. All efforts were made to minimize animal suffering and to reduce the number of animals used.

### Open Field Test

The open field test (OFT) was performed to evaluate locomotion activity and anxiety-like behavior as described previously (Sun et al., 2016). The open field apparatus consisted of a gray Plexiglas box (50-cm long × 50-cm wide × 40-cm high), which was divided into 16 virtual squares (12 peripheral squares and 4 central squares). The light source was 1.8 m above the ground. The light intensity was 50 W. Briefly, mice were allowed to explore the arena for 5 min freely. Time spent in the central squares, distance in the central squares, numbers of entries, and the total distance traveled were recorded and analyzed by the video tracking system of SMART 3.0 (Panlab Harvard Apparatus, Spain). After each test, the open-field arena was cleaned with 10% bleach and 75% ethanol (Davalli et al., 2016; Fougere et al., 2017; Hekmatimoghaddam et al., 2017; Liu E. et al., 2017; Costa-Ferreira et al., 2019; Zhou et al., 2019).

### Elevated Plus-Maze Test

The elevated plus-maze (EPM) test was used for assessing anxiety-like behavior (Rice et al., 2019). An elevated plus-shaped maze (50 cm above the floor) consisted of a central platform (5-cm long × 5-cm wide), two open arms (30-cm long × 5-cm wide), and two closed arms (30-cm long × 5-cm wide × 15-cm high). The open and close arms were situated opposite to each other. In the testing session, each mouse was placed in the central platform facing one of the open arms and allowed to explore freely for 5 min in a dim room. The percentage of open arm entries was calculated as the number of open-arms entries divided by total open and closed arms entries. The percentage of time spent in open arms was calculated as time spent in the open arms divided by total time (Franco et al., 2017; Klinger et al., 2019).

### Forced Swimming and Tail Suspension Tests

Forced swimming and tail suspension tests (FST and TST) were used to measure depression-like behaviors by recording the immobility time of animals. In the FST (Inestrosa and Varela-Nallar, 2014), the mouse was placed individually into the transparent beakers (20-cm diameter by 25-cm height) for 6 min with a water depth of 15 cm at 24–25°C. The duration of cumulative immobility was recorded during the last 4 min within the 6-min testing period. The immobility was identified as the mouse floated in the water without struggling. For the tail suspension testing (Wadhwa et al., 2018), mice were suspended through the taped and affixed approximately 1 cm from the tip of the tail, 50 cm above the floor. All mice were suspended for 6 min in each session, and the immobility period was recorded in the last 4 min of the testing period. Mice were considered to be immobile only when they were passive and completely stationary. The experimental procedures were recorded by digital video-camera.

### Novel-Object Recognition Test

The novel-object recognition (NOR) test was conducted in a square gray Plexiglas test box (50-cm long × 50-cm wide × 40-cm high) to test learning and memory (Lueptow, 2017). Animals were habituated to the arena for 5 min every day for 2 days before the test. Each mouse was allowed to explore two identical objects (A1 + A2) placed in the arena for 5 min. Three trials were conducted with an inter-trial interval of 15 min. Animals were kept in cages until the next trial at the intervals. Recognition index from the third trial during training session was used to rule out mouse preference for two objects. Mice were put back to the center of the arena and explore for another 5 min with a novel object (B; replacing A2) at 3 and 24 h after the third trial. Watching, licking or touching the object with forepaws and facing the object at the distance of approximately 2 cm were considered as object exploration. Behaviors were recorded and analyzed by the video tracking system of Smart 3.0 (Panlab Harvard Apparatus, Spain). Recognition index was calculated as the investigation time novel object / (investigation time novel object + investigation time familiar object) (Lueptow, 2017; Costa-Ferreira et al., 2019; Zhou et al., 2019).

### Y-Maze Spontaneous Alternation Test

Spatial working memory was tested in the Y-maze spontaneous alternation task (Liu E. et al., 2017). Spontaneous alternation performance was assessed by a gray Plexiglas 3-arm Y-maze (each arm: 30-cm long × 7-cm wide × 15-cm high) under weak light conditions. Mice were randomly placed in one of the three arms and allowed to explore freely for 5 min. The number and the sequence of the visited arms were recorded. The number of arm entries and the percentage of spontaneous alternation [number of alternation / (number of total arm entries-2) × 100%] was calculated.

### Morris Water Maze Test

Morris water maze (MWM) was another approach to evaluate spatial learning in rodents, as described previously (Vorhees and Williams, 2006; Xu et al., 2018). Mice were trained in a water maze (120 cm in diameter, 50 cm in depth) filled with water (25 cm in depth,23 ± 0.5°C). The pool was divided into four virtual quadrants, each with a cue to assist the mice to find the hidden platform (8.5 cm in diameter, 24 cm in height) that was submerged 1 cm under the water in the middle of one of the four quadrants. Each mouse was placed in a quadrant other than where the hidden platform was located, facing the wall of the pool, and was given 60 s to find the platform and 15 s to stay on it. Animals that did not find the platform were gently guided and placed on it during the learning test. Mice were trained for five consecutive days (three trials per day with an inter-trial interval of 20 min), and mean latency (time taken for the mouse to find the platform) from three trails per day was calculated. At 1 or 24 h after the training, the hidden platform was removed and learning was assessed through measuring the latency taken for the mouse to find the place where the hidden platform was located before and the number of crossing this place within 60 s (Klinger et al., 2019; Zhou et al., 2019).

### Mechanical Test

Paw withdrawal frequencies in response to mechanical stimuli were tested as described before (Xu et al., 2018). Briefly, each mouse was placed individually in a Plexiglas chamber on an elevated mesh. Two calibrated von Frey filaments (0.07 g and 0.4 g; Stoelting, Kiel, WI, United States) were applied to the hind paw for approximately 1 s, respectively. Each stimulation was repeated ten times to both hind paws. The occurrence of paw withdrawal in each of these ten trials was expressed as a percent response frequency [(number of paw withdrawals/10 trials) × 100 = % response frequency].

### Thermal Test

Paw withdrawal latencies to noxious thermal stimulation were examined as previously described (Porsolt et al., 1978). Each mouse was placed in a Plexiglas chamber on a glass plate. Radiant heat from the Plantar Test Instrument (Ugo Basile 37370, Italy) was applied by aiming a beam of light through the glass plate to the middle of the plantar surface of each hind paw. When the mouse lifted its foot, the light beam was cut off. The length of time between the start of the light beam and the foot lift was defined as the paw withdrawal latency. Each trial was repeated five times at 5-min intervals for each side. A cut-off time of 20 s was used to avoid tissue damage to the hind paw.

### RNA Extraction

On the final day, the mice were sacrificed immediately after behavioral tests. The anterior cingulate cortex (ACC), hippocampus and amygdala were collected in the tubes containing RNAlater (Ambion, Austin, TX, United States). Total RNA was extracted using the miRNeasy kit with on-column digestion of genomic DNA (QIAGEN, Valencia, CA, United States). RNA concentration was measured using the NanoDrop 2000 Spectrophotometer (Thermo Figher Scientific, Wilmington, DE, United States). The ratios of A260/280 nm were between 1.97 and 2.08 in all samples. RNA integrity was assessed using RNA Nanochips in an Agilent 2100 Bioanalyzer (Agilent Technologies, Santa Clara, CA, United States). RNA integrity number (RIN) was between 7.5 and 8.4.

### RNA Sequencing

Three biological replicates (three mice) from each age group were used in the RNA-seq analysis. Total RNA extracted above was subjected to rRNA depletion by Ribo-Zero rRNA Removal (Human/Mouse/Rat) Kit (Illumina, San Diego, CA, United States). Strand-specific RNA libraries were prepared using TruSeq Stranded Total RNA Sample Preparation Kit (Illumina) without poly-A selection. All assays were performed according to the manufacturer’s instructions. Sequencing was carried out at the Illumina Nova6000 plate High Output Model (Illumina, San Diego, CA, United States) (Hrdlickova et al., 2017), in a 2 × 150 bp paired-end configuration, with a total of more than 2,666 M reads per lane (at least 40 M reads per sample). All sequencing data are available in NCBI database (accession number: SRP271007).

### Bioinformatics Analysis

Nine samples from ACC, hippocampus, and amygdala (three repeats/region) were subjected to multiplexing, sequencing, differential gene expression, and transcript expression analysis. Briefly, the sequences were first trimmed in quality using trimmomatic-0.32 (Minimal length 50 bp, leading and trailing Phred Q 30). The resulting sequencing data were then mapped to the musculus genome sequence version GRCm38.72, downloaded from ENSEMBLE. Gene hit counts and reads per kilobase per million mapped reads (RPKM) were calculated for each gene to determine expression levels. DEGs and the genes with differentially expressed isoforms were filtered to *P*-value 0.05 and log base twofold change (LFC) ≥1 or ≤−1. The above RNA-Seq analyses, including mapping, read counts and expression analysis, were carried out within the CLCbio software environment (CLC Genomics Workbench 7.0.2, CLC genomics Server). Mapped reads were visualized on the UCSC browser using bigwig files converted from bam files. The heatmap was generated using Heatmaper^1^. To analyze the functions of DEGs and the genes with differentially expressed isoforms, we used GeneCards database^2^ and CTD database^3^ to map them with depression and anxiety in the ACC and amygdala as well as with cognitive dysfunction in hippocampus. They were also mapped to inflammation, apoptosis, oxidative stress, synaptic plasticity, glutamate-receptor pathway, and DNA methylation.

### Quantitative Real-Time RT-PCR

Total RNA was reverse-transcribed using the ThermoScript reverse transcriptase (Invitrogen) according to the manufacturer’s instructions with the oligo (dT) primers. cDNA was amplified by real-time PCR using the primers listed in Supplementary Table 1 (Integrated DNA Technologies, Coralville, IA, United States). Each sample was run in triplicate in a 20 μl reaction with 250 nM forward and reverse primers and 10 μl of Advanced Universal SYBR Green Supermix (Bio-Rad Laboratories, Hercules, CA, United States). Reactions were performed in a BIO-RAD CFX96 real-time PCR system. The cycle parameters were set as follows: an initial 3 min incubation at 95°C, followed by 40 cycles of 95°C for 10 s, 60°C for 30 s, and 72°C for 30 s. Ratios of mRNA levels from 12- or 24-month old mice to mRNA levels from 2-month old mice were calculated using the ΔCt method (2-ΔΔCt) at a threshold of 0.02. All data were normalized to an internal housekeeping gene *Gapdh* (Wen et al., 2020).

### Functional Enrichment Analysis of Differentially Expressed Genes

For biological pathway analysis, approximately 634, 453, and 884 DEGs respectively from ACC, amygdala, and hippocampus of the 12-month old mice and 1078, 1015, and 1054 DEGs, respectively from ACC, amygdala, and hippocampus of the 24-month old mice were selected based on significantly differential changes in their expression as compared with the 2-month old mice. Panther Classification System database system was used to categorize the differential expression genes. (© 2015, Paul Thomas^4^). To comprehensively analyze the functions of the DEGs in the ACC, amygdala and hippocampus, all DEGs (including the upregulated genes and downregulated genes) were analyzed using the gene ontology enrichment analysis from the Database for Annotation, Visualization, and Integrated Discovery (DAVID^5^).

### Protein-Protein Interaction Network Construction

STRING (version: 11.0) database^6^ was used to predict the interaction between the proteins encoded by the DEGs. The protein–protein interaction (PPI) network was built using Cytoscape software (version: 3.6.0^7^) (Liu et al., 2019). The connection degree of each node was calculated through the centiscape plugin, and the first 50 genes that were selected based on connection degree were used to construct networks. Nodes were excluded from the network due to interaction with other nodes. The size of the node was determined by the degree of connection degree in the network, and use red, blue, and purple colors to mark the genes related to inflammation, apoptosis, and both inflammation and apoptosis, respectively.

### Correlational Analysis Between mRNA Levels of DEGs and Mouse Behaviors

Three DEGs were selected from ACC, amygdala and hippocampus, respectively, of 2- and 12-month old mice according to their scores between the genes and depression, anxiety and cognitive dysfunction based on the GeneCards database^2^ and CTD database^3^. A correlation analysis between the expressional levels of these DEGs from 2- and 12-month old mice and behavioral scores from these corresponding mice was performed via the Pearson’s method using the SPSS Software (IBM SPSS Statistics 21.0).

### Statistical Analysis

All data were collected randomly and expressed as mean ± SEM. The data were statistically analyzed with two-tailed, unpaired Student’s *t*-test or one- or two-way ANOVA with repeated measures. When ANOVA showed a significant difference, the pairwise comparison between means was tested by the *post hoc* Tukey method. *P*-values less than 0.05 were considered statistically significant.

## Results

### General Appearance and Behaviors in Aging Mice

As shown in Supplementary Figure 1, the body weights of 12- and 24-month old mice were increased by 1.24-fold and 1.32-fold, respectively, as compared with 2-month old mice. Additionally, there were differences in the general appearance among these three groups of mice. The 12- and 24-month old mice exhibited some aging appearances, including slight body bending with hair loss and lacking luster. The 24-month old mice displayed lightly unstable gait and reduced spontaneous activity. There were no visible differences in the gait and spontaneous activity between the 12- and 2-month old mice.

### Impaired Cognitive and Memory Abilities in Aging Mice

**FIGURE 1:**
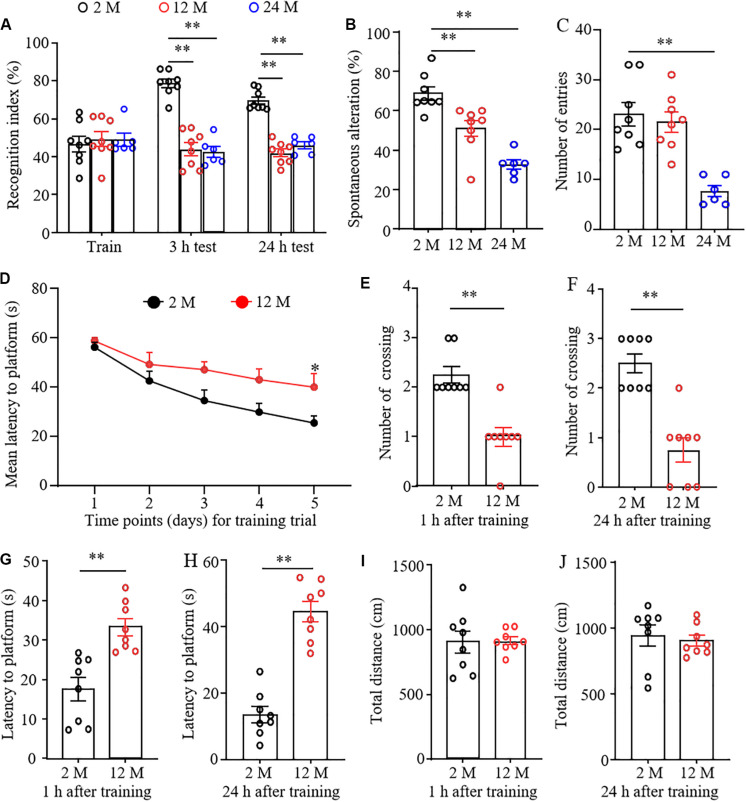
The cognitive dysfunction in aging mice. *n* = 8 for 2- or 12-month mice, *n* = 6 for 24-month mice. Data were shown as mean ± SEM. **(A)** The recognition indexes were significantly reduced in 12- and 24-month old mice as compared to 2-month old mice at 3 and 24 h after the training session in the novel object recognition test. ^∗∗^*P* < 0.01 versus the corresponding 2-month old mice by two-way ANOVA with repeated measures followed by *post hoc* Tukey test. **(B)** The percentages of spontaneous alterations were significantly reduced in 12- and 24-month old mice as compared to 2-month old mice in the Y maze test. ^∗∗^*P* < 0.01 versus the corresponding 2-month old mice by one-way ANOVA with repeated measures followed by *post hoc* Tukey test. **(C)** The number of arm entries was significantly reduced in 24-month old mice, but not in 12-month old mice in the Y maze test. ^∗∗^*P* < 0.01 versus the corresponding 2-month old mice by one-way ANOVA with repeated measures followed by *post hoc* Tukey test. **(D)** The learning curve was examined for 5 days in the Morris water maze test. ^∗^*P* < 0.05 versus 2-month old mice at the corresponding time point by two-way ANOVA with repeated measures followed by *post hoc* Tukey test. **(E,F)** Platform crossing times in the probe trial of the MWM test at 1 **(E)** and 24 h **(F)** after the training session. ^∗^*P* < 0.01 versus 2-month old mice by two-tailed unpaired Student’s *t*-test. **(G,H)** The average escape latency to reach the previous platform location was examined at 1 **(G)** and 24 h **(H)** after the training session. ^∗∗^*P* < 0.01 versus 2-month old mice by two-tailed unpaired Student’s *t*-test. **(I,J)** Total distance of movement was examined at 1 **(I)** and 24 h **(J)** after the training session. Two-tailed unpaired Student’s *t*-test.

Cognitive and memory abilities in three groups of mice were observed by novel object recognition (NOR), Y-maze spontaneous alternation, and MWM tests. In the NOR test, the mice from 2-, 12-, and 24-month old did not exhibit any differences in basal precognitive behavior in the training session (Figure 1A). However, 3 and 24 h later after training, 12- and 24-month old mice displayed a significantly reduced recognition in exploring new objects as compared with the 2-month old mice. Consistently, the Y-maze test showed that the percentages of spontaneous alternation were robustly decreased in 12- and 24-month old mice compared with those of 2-month old mice (Figure 1B), indicating the decline in spatial memory. In addition, a significant reduction in the total number of arm entries was found in 24-month old mice, but not in 12-month old mice (Figure 1C). This indicates that 24-month old mice may have abnormal locomotor activity. In the MWM test, 12-month old mice displayed a longer latency to reach the platform compared to 2-month old mice in the 5th trial in the learning curve (Figure 1D). Spatial and working memory was further determined by removing the platform. As shown in Figures 1E–H, 12-month old mice exhibited the significantly lower numbers of crossing the place where the escape platform was located previously and the prolonged escape latencies at 1 and 24 h after training, suggesting the worse memory retention in 12-month old mice. As expected, we found that the total distances traveled at 1 and 24 h probe trials did not differ between 2- and 12-month old mice (Figures 1I,J), indicating that the performance differences observed above did not result from the changes in overall activity. The 24-month old mice were not employed in the MWM test considering the high risk of this test due to the reduced spontaneous activity and impaired locomotor function (see below) in these mice.

### Depression- and Anxiety-Like Behaviors in Aging Mice

**FIGURE 2:**
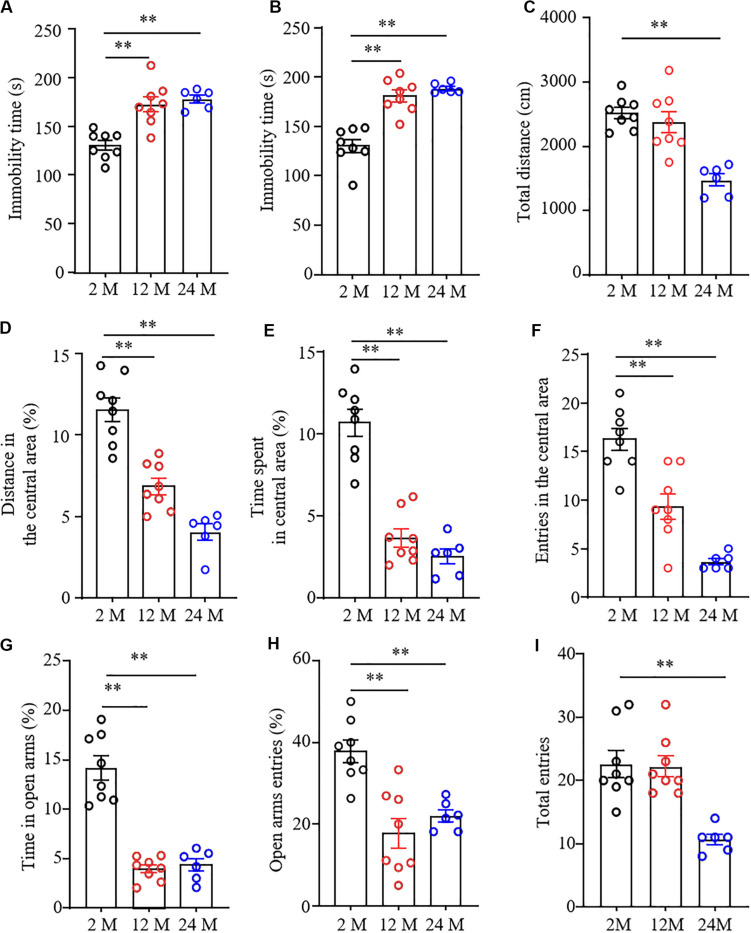
Depression- and anxiety-like behavior in aging mice. *n* = 8 for 2- or 12-month mice, *n* = 6 for 24-month mice. Data were shown as mean ± SEM. **(A,B)** The immobility time was significantly increased in 12- and 24-month old mice in the forced swimming test (FST) **(A)** and tail suspension test (TST) **(B)** as compared to 2-month old mice. ^∗∗^*P* < 0.01 versus 2-month old mice by one-way ANOVA with repeated measures followed by *post hoc* Tukey test. **(C)** The total distance of 24-month-old mice in the open field test was less than that of 2-month-old mice. ^∗∗^*P* < 0.01 versus 2-month old mice by one-way ANOVA with repeated measures followed by *post hoc* Tukey test. **(D–F)** The percentage of distance in the central area **(D)**, the time spent in the central area **(E)**, and the entries in the central area **(F)** were significantly reduced in 12- and 24-month old mice in the open field test as compared to 2-month-old mice. ^∗∗^*P* < 0.01 versus 2-month old mice by one-way ANOVA with repeated measures followed by *post hoc* Tukey test. **(G,H)** The percentages of the time spent in open arms **(G)** and open arms entries **(H)** were both significantly reduced in 12- and 24-month old mice as compared to 2-month old mice in the elevated plus maze test. ^∗∗^*P* < 0.01 versus 2-month old mice by one-way ANOVA with repeated measures followed by *post hoc* Tukey test. **(I)** The total arm entries of 24-month old mice were less than those of 2-month old mice. ^∗∗^*P* < 0.01 versus 2-month old mice by one-way ANOVA with repeated measures followed by *post hoc* Tukey test.

Forced swimming tests and tail suspension tests are the classical methods to screen the rapid behavioral changes in stressful situations. Immobility time was used as an indicator of depression in these tests. As shown in Figures 2A,B, immobility times in both 12- and 24-month old mice were significantly prolonged as compared with 2-month old mice, demonstrating that the aging mice were more likely to give up struggling in both tests due to despair.

To determine the anxiety-like behavior in aging mice, we performed the open field and EPM tests. In the OFT, we found that the overall distance traveled was conspicuously decreased in 24-month old mice, but not in 12-month old mice (Figure 2C), suggesting the impaired locomotor activity in 24-month old mice. Further observations showed the reduced exploration activity in 12- and 24-month old mice evidenced by the considerable reductions in distance traveled and time spent in the center area (Figures 2D,E), as well as in numbers of entering the central area (Figure 2F). Similar to whatever we observed in OFT, the EPM test showed that both the percentage of time spent in the open arms (Figure 2G) and the percentage of open arm entries were much lower in 12- and 24-month old mice than those in 2-month old mice (Figures 2G,H). As expected, the significantly decreased number of total entries to both open and closed arms in 24-month old mice, but not 12-month old mice, was seen (Figure 2I), further demonstrating impaired motor function only in 24-month old mice. Taken together, these findings indicate the depression- and anxiety-like behaviors in aging mice.

### Basal Nociceptive Behavioral Responses in Aging Mice

**FIGURE 3:**
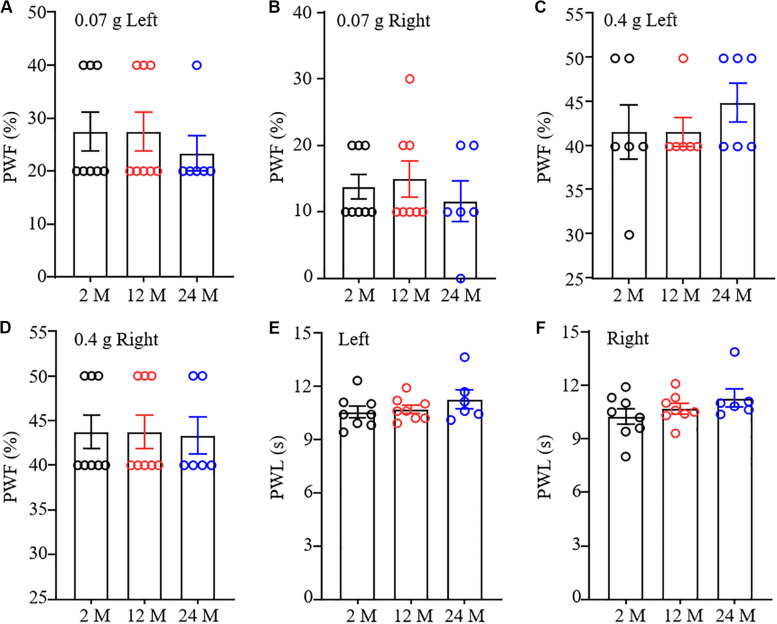
Nociceptive response in different ages of mice. Paw withdrawal frequencies (PWF) in response to 0.07 g **(A,B)** and 0.4 g **(C,D)** von Frey filaments and paw withdrawal latency (PWL) on response to heat stimulation **(E,F)** on the left **(A,C,E)** and right **(B,D,F)** sides. *n* = 8 for 2- or 12-month mice, *n* = 6 for 24-month mice. Data were shown as mean ± SEM. One-way ANOVA with repeated measures followed by *post hoc* Tukey test.

To understand comprehensive phenotypes with aging, we examined basal nociceptive behavioral responses to mechanical and thermal stimuli in these three different ages of mice. As shown in Figure 3A–D, there were no significant differences in paw withdrawal frequencies in response to 0.07 g and 0.4 g von Frey filament stimuli among 2-, 12-, and 24-month old mice in both hind paws, although 24-month old mice exhibited a decreased tendency in response to 0.07 g von Frey filament stimulation. In addition, the mice from these three different ages showed similar paw withdrawal latencies to heat stimulation on both sides (Figures 3E,F).

### RNA-seq and Genome-Wide Read Mapping in ACC, Hippocampus, and Amygdala of Aging Mice

**FIGURE 4:**
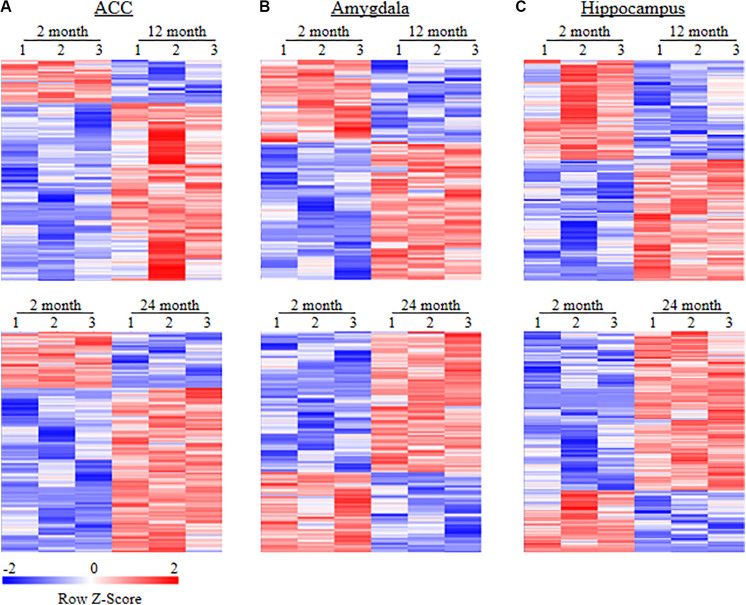
Heatmaps of differentially expressed genes (DEGs) in the ACC **(A)**, amygdala **(B)** and hippocampus **(C)** from 2-month old mice versus 12-month old mice (top) or 24-month old mice (bottom). Colors in the heatmaps indicate the Row *Z*-score among the different data sets. High expression is shown by the red color spectrum, and low expression is shown by the blue. *N* = 3 biological repeats (3 mice)/age.

**FIGURE 5:**
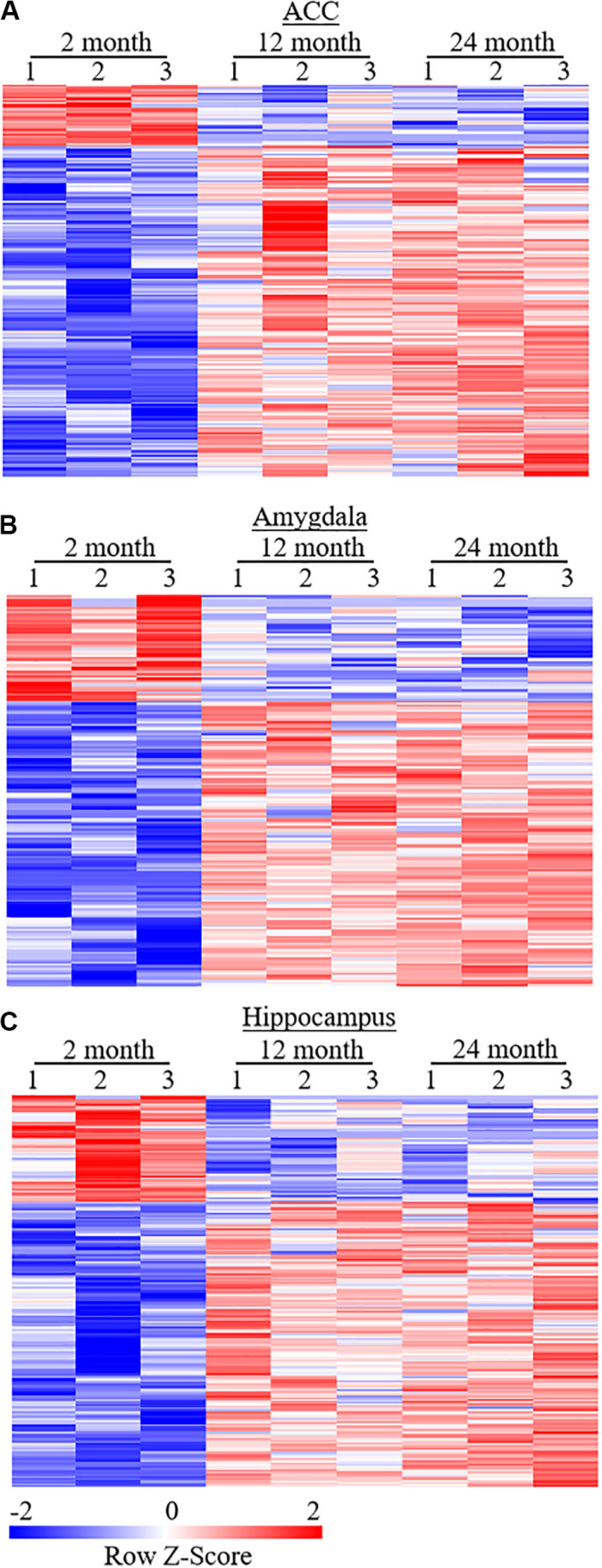
Heatmaps of differentially expressed genes (DEGs) in the ACC **(A)**, amygdala **(B)**, and hippocampus **(C)** from both 12- and 24-month old mice versus 2-month old mice. Colors in the heatmaps indicate the Row *Z*-score among the different data sets. High expression is shown by the red color spectrum, and low expression is shown by the blue. *N* = 3 biological repeats (3 mice)/age.

To examine whether behavioral changes observed above are associated with DEGs in brain regions of aging mice, we carried out the RNA-sequencing analysis to define the gene expression profiles in the ACC, hippocampus, and amygdala from these three different ages of mice. In order to visualize the collected data of DEGs, we generated the clustered heatmaps to show the changes in the genes by color intensity. As shown in Figure 4, compared to the gene expression profile from 2-month old mice, about 634 (510 increased and 124 decreased) and 1078 (805 increased and 273 decreased) DEGs in ACC, 453 (283 increased and 170 decreased) and 1015 (646 increased and 369 decreased) DEGs in the amygdala and 884 (485 increased and 399 decreased), and 1054 (759 increased and 295 decreased) DEGs in hippocampus were detected in 12- and 24-month old mice, respectively. Further analysis revealed that approximately 222 (187 increased and 35 decreased) DEGs in ACC, 171 (125 increased and 46 decreased) DEGs in the amygdala and 315 (229 increased and 86 decreased) DEGs in hippocampus were seen in both 12- and 24-month old mice (Figure 5).

**FIGURE 6:**
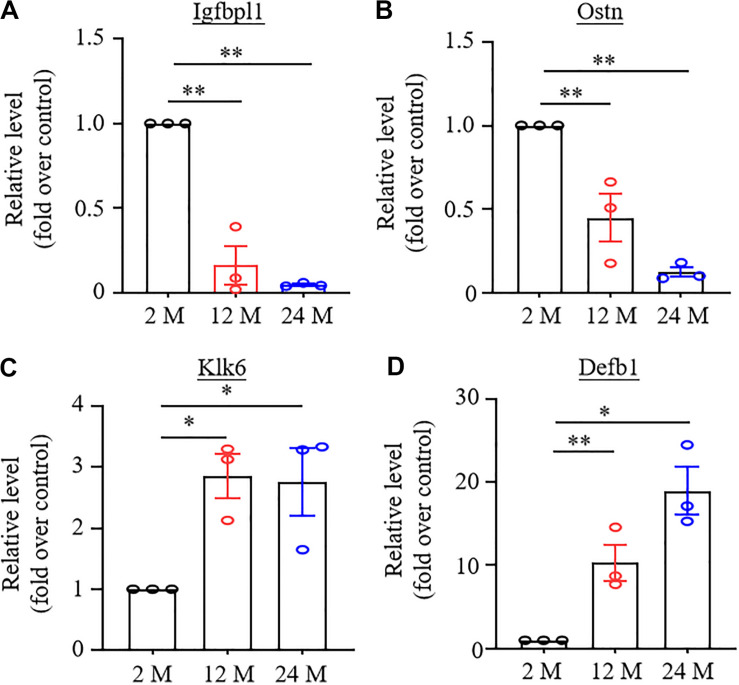
Verification of the changes in expression of some differentially expressed genes using real-time reverse transcription polymerase chain reaction (RT-PCR) analysis. *N* = 3 biological repeats (3 mice)/age. Data were shown as mean ± SEM. **(A,B)** Expression of *Igfbpl1* mRNA in the hippocampus **(A)** and *Ostn* mRNA in ACC **(B)** were significantly reduced in both 12- and 24-month old mice as compared to 2-month old mice. ^∗∗^*P* < 0.01 versus the corresponding 2-month old mice by one-way ANOVA with repeated measures followed by *post hoc* Tukey test. **(C,D)** Expression of *Klk6* mRNA in amygdala **(C)** and *Defb1* mRNA in ACC **(D)** were significantly increased in 12- and 24-month old mice as compared to 2-month old mice.^ *^P < 0.05, ^∗∗^*P* < 0.01 versus 2-month old mice by one-way ANOVA with repeated measures followed by *post hoc* Tukey test.

To validate the results achieved from the RNA-sequencing analysis, we randomly selected four DEGs, which were changed significantly (LFC ≥2.2 or ≤−2.2), in both 12- and 24-month old mice, for quantitative RT-PCR assay. As expected, the amount of *Igfbpl1* RNA in the hippocampus and the amount of *Ostn* RNA in the ACC were markedly reduced (Figures 6A,B), while the level of *Klk6* RNA in the amygdala and the level of *Defb1* RNA in the ACC were significantly increased in both 12- and 24-month old mice (Figures 6C,D).

### Functional Enrichment Analysis of the Differentially Expressed Genes in Aging Mice

**FIGURE 7:**
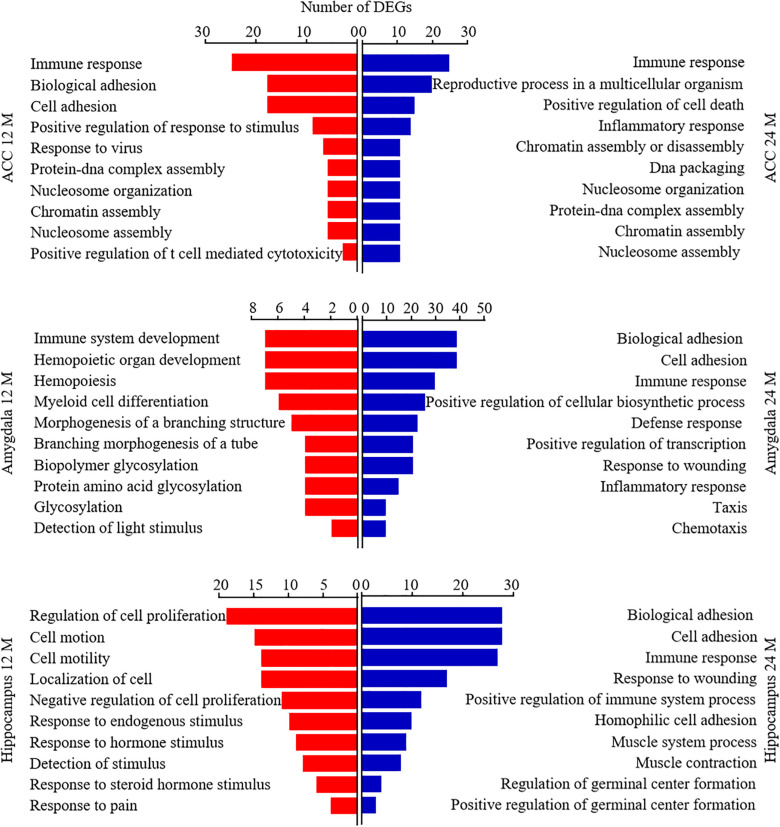
Functional enrichment analysis of the differentially expressed genes (DEGs) in aging mice. Analysis of the Gene Ontology database showed top 10 biological processes of the DEGs in the ACC (top), amygdala (middle), and hippocampus (bottom) from 12- and 24-month old mice according to the *P*-value of biological process. The DAVID database was used to do the GO enrichment analysis. Red and blue color bars represent 12- and 24-month old mice, respectively.

Database for Annotation, Visualization, and Integrated Discovery bioinformatics database was used to analyze the Gene Ontology analysis and categorize the DEGs based on the distinct processes. Figure 7 showed the analysis of DEGs for biological processes. The DEGs in the ACC from both 12- and 24-month old mice were enriched highly in the immune response. The DEGs in the amygdala from 12-month old mice were enriched predominantly in immune system development, hemopoietic organ development, and hemopoiesis. The DEGs in both amygdala and hippocampus from 24-month old mice were enriched mainly in biological adhesion, cell adhesion, and immune response. The DEGs in the hippocampus from 12-month old mice were enriched largely in the regulation of cell proliferation, cell motion, cell motility, and localization of cell. We also categorized these DEGs according to their molecular functions (Supplementary Figure 2). The DEGs in ACC from 12-month old mice and in the hippocampus from both 12- and 24-month old mice were enriched mostly in calcium ion binding function. The DEGs in the amygdala from 24-month old mice were enriched predominantly in carbohydrate-binding function. The DEGs in the amygdala from 12-month old mice were enriched mainly in enzyme inhibitory activity, peptidase inhibitory activity, carbohydrate-binding, and endopeptidase inhibitory activity functions. The DEGs in ACC from 24-month old mice were enriched mainly in peptide binding, peptide receptor activity, cytokine activity, and hormone activity functions. Finally, we divided these DEGs according to their cellular components (Supplementary Figure 3). In both 12- and 24-month old mice, the DEGs in ACC were enriched predominantly in the plasma membrane and extracellular region, the DEGs in the amygdala mainly in the extracellular region and the DEGs in hippocampus mostly in intrinsic and integral to the membrane.

### Depression, Anxiety, and Cognitive Dysfunction-Associated DEGs in Aging Mice

To further analyze the relationship between DEGs and behavioral changes with aging, we used the GeneCards database and CTD database to search cognitive dysfunction, depression, and anxiety-related genes. Approximately 2000 genes were selected according to the relevance score as data sets for the subsequent analysis. Given that ACC and amygdala play an important role in the genesis of depression and anxiety and that hippocampus is a key region associated with cognitive dysfunction^8–13^, we compared the depression- and anxiety-related gene data sets with the DEGs in the ACC and amygdala, and the cognitive dysfunction-related gene data set with the DEGs in the hippocampus. About 47 and 96 depression-related DEGs in the ACC and 25 and 103 depression-related DEGs in the amygdala were found in 12- and 24-month old mice, respectively (Supplementary Material 1). Approximately 205 and 124 anxiety-related DEGs in the ACC and 110 and 128 anxiety-related DEGs in the amygdala were seen in 12- and 24-month old mice, respectively (Supplementary Material 1). Finally, there were about 125 and 128 cognitive dysfunction-related DEGs in the hippocampus of 12- and 24-month old mice, respectively (Supplementary Material 1).

Given that the depression/anxiety-related behaviors and cognitive dysfunction were associated with inflammation, apoptosis, oxidative stress, synaptic plasticity, glutamate receptor pathway, and DNA methylation (Makhija and Karunakaran, 2013; Sheng and Erturk, 2014; Cai et al., 2015; Januar et al., 2015), we further analyzed the functions of the DEGs in the ACC, amygdala and hippocampus. All depression/anxiety/cognitive-related DEGs in three brain regions were associated with inflammation in both 12- and 24-month old mice (Supplementary Table 2). In addition, among the depression-related DEGs in the ACC, about 96, 87, 26, 66, and 46% from 12-month old mice and about 89, 84, 36, 67, and 92% from 24-month old mice were associated with apoptosis, oxidative stress, synaptic plasticity, glutamate receptor pathway, and DNA methylation, respectively (Supplementary Table 2). Among the depression-related DEGs in the amygdala, approximately 92, 80, 36, 72, and 88% from 12-month old mice and approximately 87, 88, 41, 71, and 91% from 24-month old mice were associated with apoptosis, oxidative stress, synaptic plasticity, glutamate receptor pathway, and DNA methylation, respectively (Supplementary Table 2). Among the anxiety-related DEGs in AAC, about 44, 36, and 63% from 12-month old mice and approximately 49, 42, and 60% from 24-month old mice were associated with oxidative stress, glutamate receptor pathway, and DNA methylation, respectively (Supplementary Table 2). Among the anxiety-related DEGs in amygdala, about 23, 30, and 56% from 12-month old mice and approximately 64, 46, and 67% from 24-month old mice were associated with oxidative stress, glutamate receptor, and DNA methylation, respectively (Supplementary Table 2). Among the cognitive dysfunction-related DEGs in the hippocampus, about 78, 66, 34, 53, and 75% from 12-month old mice and about 79, 70, 34, 61, and 73% from 24-month old mice were associated with apoptosis, oxidative stress, synaptic plasticity, glutamate receptor pathway, and DNA methylation, respectively (Supplementary Table 2).

### Depression, Anxiety and Cognitive Dysfunction-Associated Genes With Differentially Expressed Isoforms in Aging Mice

We also used the same strategy to analyze the relationship between the genes with differentially expressed isoforms and behavioral changes with aging. About 525 and 507 depression-related genes in the ACC and 448 and 564 depression-related genes in the amygdala were found in 12- and 24-month old mice, respectively (Supplementary Material 2). Approximately 1918 and 1998 anxiety-related genes in the ACC and 1706 and 1991 anxiety-related genes in the amygdala were seen in 12- and 24-month old mice, respectively (Supplementary Material 2). Finally, there were about 920 and 881 cognitive dysfunction-related genes in the hippocampus from 12- and 24-month old mice, respectively (Supplementary Material 2).

Further analysis revealed that all of these depression/anxiety/cognitive-related genes with differentially expressed isoforms in these three regions were associated with inflammation in both 12- and 24-month old mice (Supplementary Table 3). In addition, among the depression-related genes with differentially expressed isoforms in ACC, about 89, 80, 42, 67, and 91% from 12-month old mice and about 87, 80, 38, 64, and 87% from 24-month old mice were associated with apoptosis, oxidative stress, synaptic plasticity, glutamate receptor pathway, and DNA methylation, respectively (Supplementary Table 3). Among the depression-related genes with differentially expressed isoforms in amygdala, approximately 89, 80, 40, 65, and 88% from 12-month old mice and approximately 87, 78, 41, 65, and 89% from 24-month old mice were associated with apoptosis, oxidative stress, synaptic plasticity pathway, glutamate receptor pathway, and DNA methylation, respectively (Supplementary Table 3). Among the anxiety-related genes with differentially expressed isoforms in AAC, about 56, 39, and 71% from 12-month old mice and approximately 55, 38, and 67% from 24-month old mice were associated with oxidative stress, glutamate receptor pathway, and DNA methylation, respectively (Supplementary Table 3). Among the anxiety-related genes with differentially expressed isoforms in amygdala, about 58, 38, and 69% from 12-month old mice and approximately 57, 39, and 70% from 24-month old mice were associated with oxidative stress, glutamate receptor pathway, and DNA methylation, respectively (Supplementary Table 3). Among the cognitive dysfunction-related genes with differentially expressed isoforms in hippocampus, about 81, 71, 35, 55, and 80% from 12-month old mice and about 82, 74, 33, 54, and 80% from 24-month old mice were associated with apoptosis, oxidative stress, synaptic plasticity, glutamate receptor pathway, and DNA methylation, respectively (Supplementary Table 3).

### Establishing a PPI Network to Analyze Protein–Protein Interactions

**FIGURE 8:**
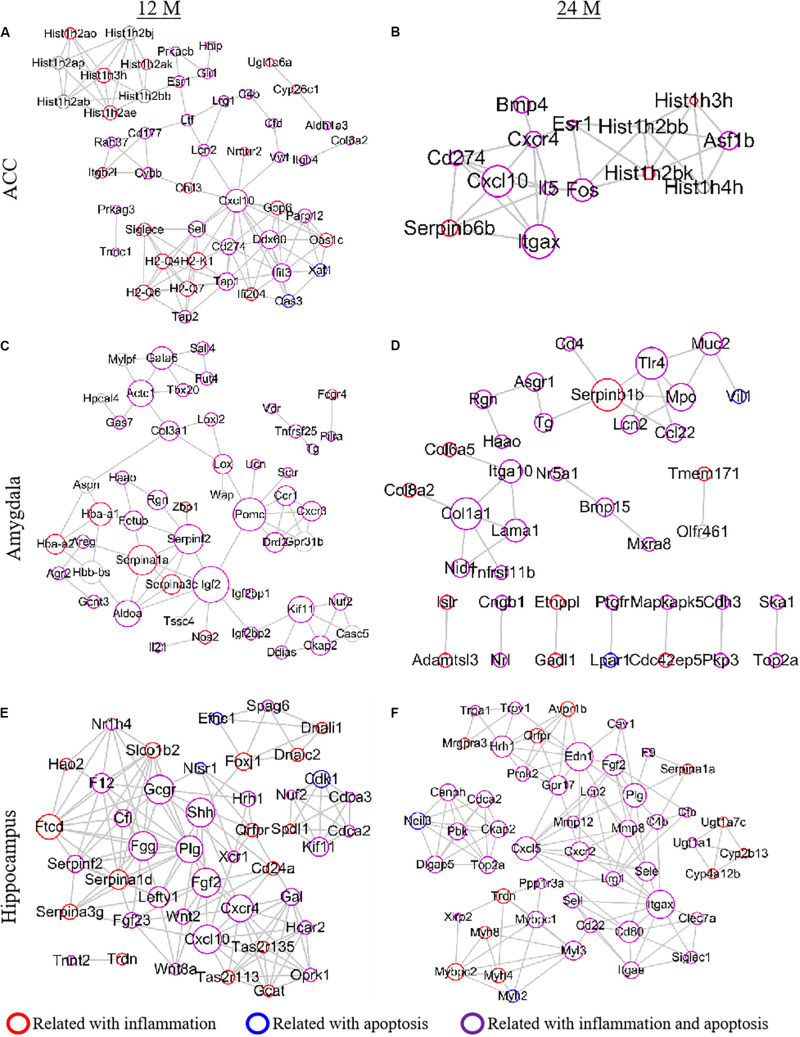
Establishment of a PPI network to analyze protein-protein interactions. Top 50 differentially expressed genes (DEGs) were selected according to the connection degree of genes in the ACC **(A,B)**, amygdala **(C,D)**, and hippocampus **(E,F)** of 12- **(A,C,E)** and 24- **(B,D,F)** month old mice. The size of the node is determined by the connection degree. The connection degree reflects the importance of the gene in the network as it represents the number of nodes connected. The red, blue, and purple circles represent the genes related to inflammation, apoptosis and both inflammation and apoptosis, respectively.

To explore the relationship between the DEGs and aging pathology, we performed the PPI network analysis using the STRING database (Figure 8). Due to the involvement of a large number of DEGs in the network, the top 50 DEGs from each region were selected to draw the PPI network according to the connection degree. A serial of networks was generated, and the hub genes were shown in the network. Cxcl10, an important chemokine, was the central node of the network in the ACC of 12- and 24-month old mice (Figures 8A,B). Serpina1a, Serpinf2, Igf2, and Pomc played a crucial role in the network of the amygdala in 12-month old mice (Figure 8C). In contrast, Serpin family and Serpinb1b were central molecules in the network of the amygdala in 24-month old mice (Figure 8D). Moreover, there were 10 genes (Gcgr, Shh, Fgg, Plg, Fgf2, Cxcr4, Cxcl10, Ftcd, Edn1, Itgax, and Cxcl5) played a critical role in the network of the hippocampus of 12- and 24-month old mice (Figures 8E,F). The network also showed that the inflammation- and apoptosis-related genes occupied a vital position in the PPI networks (Figure 8).

### Association of Several DEGs With Depression-, Anxiety-, or Cognitive Dysfunction-Related Behaviors in Aging Mice

Finally, we carried out the Pearson method to analyze the correlation between mRNA levels of three selected DEGs from the ACC/amygdala and the depression/anxiety-like behaviors or between mRNA levels of three selected DEGs from hippocampus and cognitive dysfunction. In the ACC, the levels of *Esr1, Crhr2*, and *Tgif1* mRNAs were positively associated with the immobile time of the forced swimming and TSTs (*r* = 0.848 ∼ 0.977, *P* = 0.033 ∼ 0.001; Supplementary Table 4). The amount of *Tshr* mRNA was negatively associated with the percentage of distance in central (*r* = −0.939, *P* = 0.006) and the entries in central (*r* = −0.931, *P* = 0.007) in OFT as well as the percentage of entries in open arms (*r* = −0.968, *P* = 0.001) and the percentage of time in open arms (*r* = −0.833, *P* = 0.039) in EPM test (Supplementary Table 4). The level of *Esr1* mRNA was negatively associated with the percentages of distance in central (*r* = −0.856 and *P* = 0.030) and the entries in central (*r* = −0.899, *P* = 0.015) in OFT and with the percentages of entries in open arms in EPM test (*r* = −0.909, *P* = 0.012). Interestingly, the amount of *Crhr2* mRNA was only negatively related to the entries in central in OFT (*r* = −0.876, *P* = 0.022) (Supplementary Table 4). In the amygdala, the immobile time of the tail suspension and FSTs was positively associated with the level of *Vdr* mRNA (*r* = 0.886, *P* = 0.019; *r* = 0.872, *P* = 0.023) and negatively related to the amount of *Kif1l* mRNA (*r* = −0.850, *P* = 0.032; *r* = −0.823, *P* = 0.044) (Supplementary Table 4). Unexpectedly, no significant associations were observed between *Pomc* mRNA expression and these depression-like behaviors in mice (Supplementary Table 4). In addition, the levels of *Bcl2l10, Serpinf2*, and *Piwil4* mRNAs displayed high positive correlations to the parameters indicated above in the OFT (*r* = −0.858 ∼−0.884 and 0.828 ∼ 0.944, *P* = 0.042 ∼ 0.005; Supplementary Table 4). In the EPM test, the level of Serpinf2 mRNA was positively relevant to the percentage of entries (*r* = 0.852, *P* = 0.031) and the percentage of time (*r* = 0.860, *P* = 0.028) in open arms, the amount of *Bcl2l10* was negatively associated with the percentage of entries in open arms (*r* = −0.852, *P* = 0.031) and the level of *Piwil4* mRNA was unrelated to either parameter (Supplementary Table 4). In the hippocampus, the level of *Plg* mRNA was negatively associated with the percentages of spontaneous alternation in the Y-maze spontaneous alternation tests (*r* = −0.892, *P* = 0.017), recognition in exploring new objects 3 h (*r* = −0.956, *P* = 0.003) and 24 h (*r* = −0.854, *P* = 0.030) after training in the NOR test, and the numbers of platform crossing in the MWM tests (*r* = −0.889, *P* = 0.018) (Supplementary Table 5). In contrast, the level of *Dbh* mRNA was positively related to the percentages of spontaneous alternation in the Y-maze spontaneous alternation tests (*r* = 0.884, *P* = 0.019) and recognition in exploring new objects 3 h after training in the NOR test (*r* = 0.855, *P* = 0.030) (Supplementary Table 5). The level of *Prph* mRNA was negatively associated only with the numbers of platform crossing 1 h after training in the MWM test (*r* = −0.869, *P* = 0.025) (Supplementary Table 5).

## Discussion

Despite intensive research into the molecular and biological mechanisms of aging and aging-related diseases during past decades, aging and aging-related diseases are poorly managed by current drugs. In the present study, we used a mouse model of natural aging to mimic the different aging stages in humans to search the aging symptoms-associated genes in ACC, amygdala and hippocampus. Behavior tests were first carried out to verify depression/anxiety-like behaviors and learning, memory and cognitive impairments in the 12- and 24-month old mice. Bioinformatical analyses further showed that all DEGs and the genes with differentially expressed isoforms identified in ACC, hippocampus and amygdala of two groups of aging mice were closely related to the neuro-inflammation. Our findings demonstrate that cognitive impairment and emotional disorders with aging are likely corresponding to the changes in neuroinflammation-related genes.

In the present study, we found that the mice of 12- and 24-month old showed the progressive loss in episodic-like memory with aging demonstrated by NOR assay. This observation was consistent with the previous reports that the object recognition memory seemed to be impaired as early as the age of 12 months (Belblidia et al., 2018). Moreover, in both groups of aged mice, spatial memory with aging was impaired evidenced by the decreased percentage of spontaneous alternation during the Y-maze test and by the prolonged escape latency, as well as the reduced numbers of crossing the position where the platform located in the training sessions during MWM test. Consistent with a previous study (Bach et al., 1999), 24-month old mice exhibited a decline in locomotor activity evaluated by the decrease in total distance traveled in OFT assay and reduced number of the entry in EPM test.

Although the mechanism of age-related emotional dysfunction remains controversial in many studies (Schulz et al., 2007; Malatynska et al., 2012), increasing evidence suggests that cognitive impairment in the aged population often accompanied by depression and anxiety disorders (Kuring et al., 2018) 52. Our findings demonstrated that the immobility time was increased in both forced swimming and TSTs in aging mice. However, in the OFT, there were no apparent changes in exploration activity between 2- and 12-month old mice, indicating depression-like behaviors mainly affected the later stage of the aging process. Anxiety-like behavior in aged mice was demonstrated by the decreased percentages of open arm entries and the time spent in open arms using EPM test, an observation in agreement with the clinical studies that memory loss and dementia were often accompanied by neuropsychiatric symptoms in abnormal aging patients (Peprah and McCormack, 2019).

The previous studies on age-related changes in pain sensitivity response are controversial (Yezierski, 2012). Several reports showed that pain perception is diminished in older people (Gibson and Helme, 2001; Gibson and Farrell, 2004), whereas other observations revealed the pain threshold increased in old age (Lautenbacher, 2012). Comprehensive reviews by EI Tumi et al. recently suggest that that old adult may be more sensitive to mechanically evoked pain but not heat-evoked pain than young adults (El Tumi et al., 2017). The present study showed no significant changes in basal sensitivities in response to evoked mechanical and thermal stimuli in both 12- and 24-month old mice compared to 2-month old mice, which is in line with the previous reports in the animals (Fahlstrom et al., 2012; Shoji et al., 2016; Millecamps et al., 2019; Shoji and Miyakawa, 2019). The reasons of why preclinical studies are inconsistent with clinical observations are unclear, but might be due to the inconsistent stages of the aging process between humans and animals as well as the limited measurement methods, parameters, and dimensions in animal studies.

Despite more and more attention focused on aging and age-related disorders in clinical and preclinical investigations, the underlying mechanisms remain unclear. To gain insight into the aging process and provide the potential targets, gene expression profile changes from aging mice were detected in the present study. We identified about 634 and 1078 DEGs in ACC, 453 and 1015 DEGs in the amygdala, 884 and 1054 DEGs in the hippocampus in the 12- and 24-month old mice, respectively. We also identified many genes with differentially expressed isoforms in these three brain regions in the 12- and 24-month old mice. Further function analysis of DEGs and the genes with differentially expressed isoforms revealed that many of them in the ACC and amygdala were mapped to depression- and anxiety-related genes, respectively and that a lot of them in hippocampus were mapped to cognitive dysfunction-related genes from both 12- and 24-month old mice. All of these mapped DEGs and the genes with differentially expressed isoforms were closely related to neuroinflammation. Some of them were associated with apoptosis, oxidative stress, synaptic plasticity, glutamate receptor pathway or DNA methylation. We found that Cnr2 and Tlr4 genes related to neuroinflammation were robustly upregulated in the ACC and amygdala of aging mice, suggesting that these two genes play an important role in the genesis of depression and anxiety during aging. This conclusion is strongly supported by the previous studies that showed that knockout of Cnr2 in the brain could attenuate psychomotor, depression- and anxiety-like behaviors in mice (Liu Q.R. et al., 2017) and that blocking Tlr4 could reduce the anxiety- and depression-like behaviors (Strekalova et al., 2015; Zhang et al., 2020). In addition, we also found that the other two neuroinflammation-related genes Fgf2 and Cbln1 were differentially expressed in the hippocampus of aging mice, both of which have been demonstrated to affect fear conditioning and spatial memory processes in aged animals (Hirai et al., 2005).

It should be pointed out that present study has several limitations to be considered. Firstly, although we reported significant correlations of several selected DEGs in AAC, amygdala and hippocampus with depression-, anxiety-, or cognitive dysfunction-like behaviors, these correlations do not imply causation. Thus, we should be very careful for the interpretation of our data and perform more stringent researches to examine the roles of these candidate genes in depression, anxiety or cognitive dysfunction. Secondly, the expressional levels of some selected DEGs were not significantly associated with some parameters in behavioral tests or had no correlations with depression-, anxiety-, or cognitive dysfunction-like behaviors. These non-associations/non-correlations may be attributed to low number of the samples per group (*n* = 3 repeats/group). Increasing number of the samples per group may improve correlation efficient. Thirdly, our research on depression, anxiety and cognitive dysfunction in aging focused on male mice. There have been few studies on female mice so far. However, gender-specific effect of DEGs on depression, anxiety and cognitive dysfunction in aging could exist and remains to be addressed in the future.

## Conclusion

In conclusion, we demonstrated that progressive memory impairment and emotional disorders with aging were relevant to the neuroinflammation-related DEGs and the genes with differentially expressed isoforms in ACC, amygdala and hippocampus. Our findings may uncover a new layer of aging-related transcriptional regulation and be helpful for the drug development for management of aging and age-related disorders.

## Data Availability Statement

All sequencing data are available in NCBI database (accession number: SRP271007).

## Ethics Statement

The animal study was reviewed and approved by the Animal Care and Use Committee of Zhengzhou University.

## Author Contributions

YA and Y-XT conceived the project, supervised all experiments, and edited the draft. ML, SS, JC, XM, WZ, JY, Y-XT, and YA assisted with experimental design. ML, SS, and XM took care of feeding and maintenance of old mice. ML, SS, and WC carried out behavioral tests. ML performed tissue collection and RT-PCR assay. ML, SG, and YX analyzed the data and wrote the draft of manuscript. All authors read and discussed the manuscript.

## Conflict of Interest

The authors declare that the research was conducted in the absence of any commercial or financial relationships that could be construed as a potential conflict of interest.

## Footnotes

1 http://www.heatmapper.ca/expression/

2 https://www.genecards.org/

3 http://ctdbase.org/

4 pantherdb.org

5 http://david.abcc.ncifcrf.gov/

6 https://string-db.org/cgi/

7 www.cytoscape.org/
