# Supplementary material for: Differentially Expressed Genes in the Brain of Aging Mice With Cognitive Alteration and Depression- and Anxiety-Like Behaviors
Source: Front Cell Dev Biol. 2020 Aug 28;8:814. doi: 10.3389/fcell.2020.00814 (PMC7493670; doi:10.3389/fcell.2020.00814)
Supplement: Supplementary file 3 [file Presentation_1.pdf]

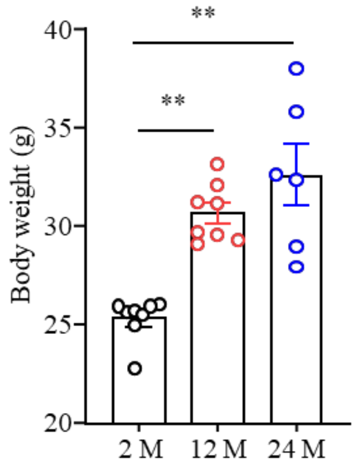

Supplementary Figure 1. Body weight changes in aging mice. The body weight of 12- and 24-month old mice was increased significantly as compared to 2-month old mice. Data were shown as mean ± SEM. *n* = 8 for 2- or 12-month mice, *n* = 6 for 24-month mice. $^{**}P < 0.01$ versus 2-month old mice by one-way ANOVA with repeated measures followed by *post hoc* Tukey test.

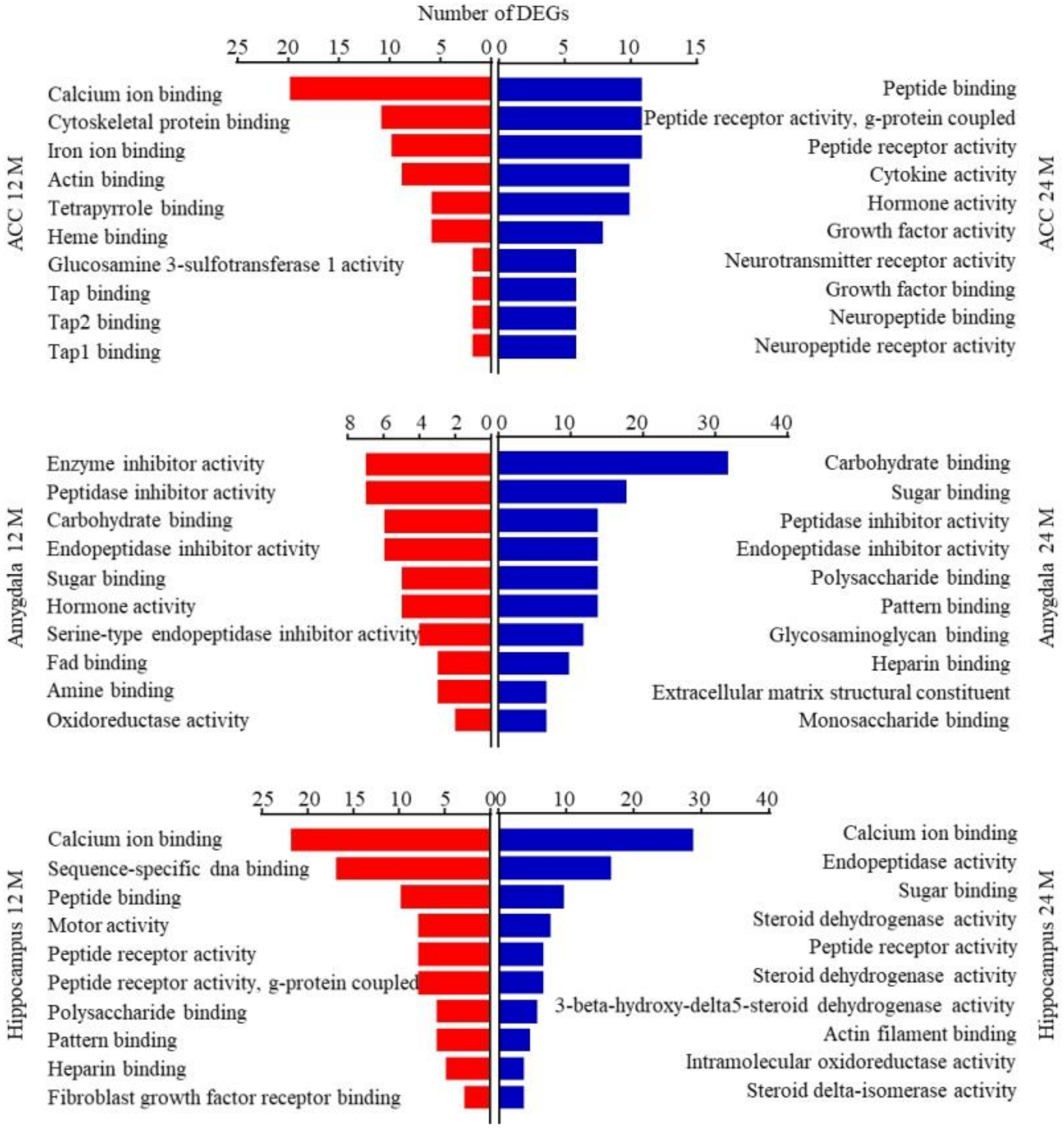

Supplementary Figure 2. Functional enrichment analysis of the differentially expressed genes in aging mice. Analysis of the Gene Ontology database showed top 10 molecular functions of the DEGs in the ACC (top), amygdala (middle), and hippocampus (bottom) from 12- and 24-month old mice according to the *p*-value of molecular process. The DAVID database was used to do the GO enrichment analysis. Red and blue color bars represent 12- and 24-month old mice, respectively.

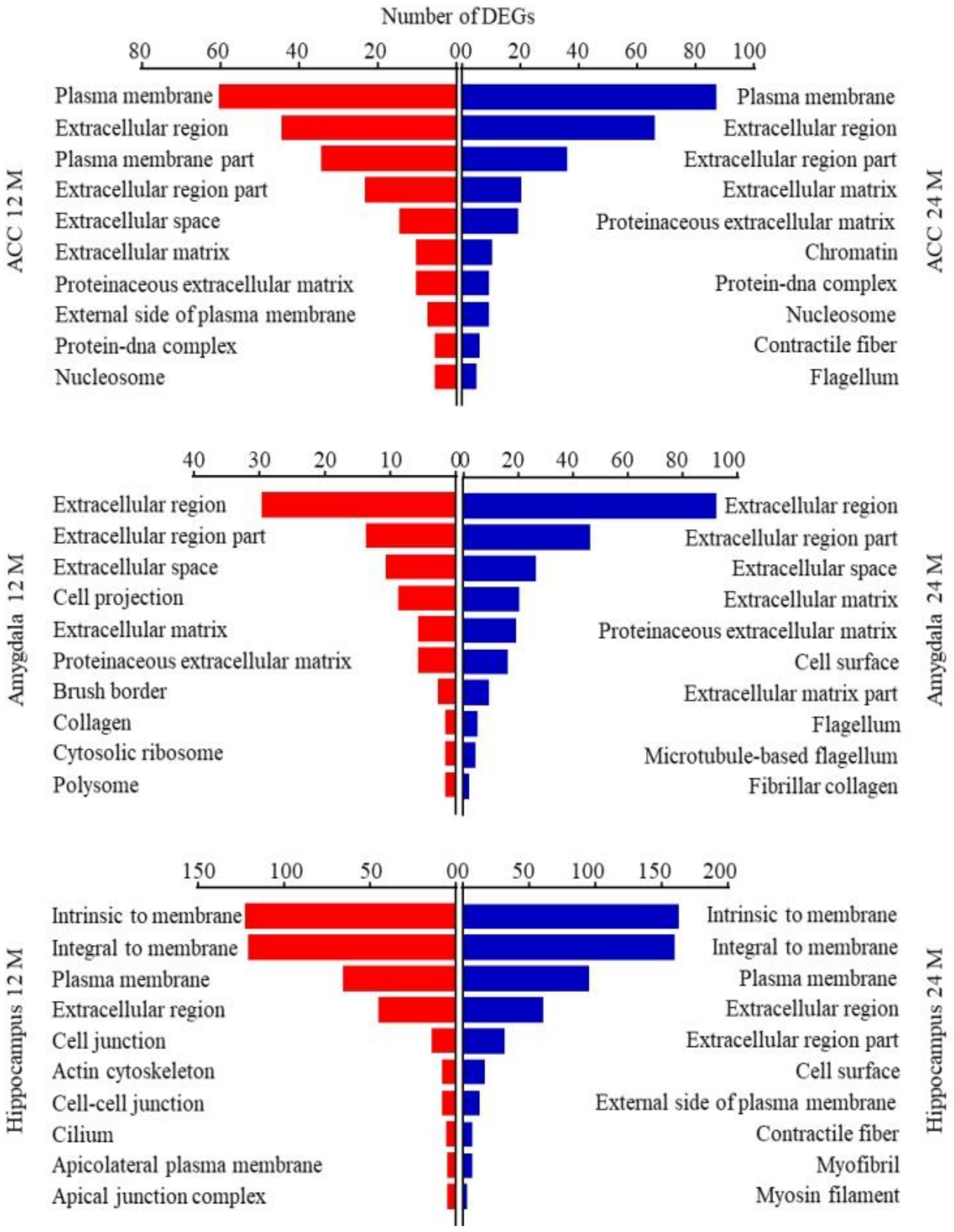

Supplementary Figure 3. Functional enrichment analysis of the differentially expressed genes in aging mice. Analysis of the Gene Ontology database showed top 10 cellular components of the DEGs in the ACC (top), amygdala (middle), and hippocampus (bottom) from 12- and 24-month old mice according to the *p*-value of cellular components. The DAVID database was used to do the GO enrichment analysis. Red and blue color bars represent 12- and 24-month old mice, respectively.

Supplementary Table 1. Primers for real-time reverse transcription polymerase chain reaction

Gene names	Primer sequences	
	Forward	Reverse
Igfbpl1	GGGACTCAAGTATTCCTTTCCTG	GCACCTGGACAGCTATATTGAC
Klk6	CCTGTGCTTGGTTCTTGCTAA	AAGTGACCTGAGGTGTAGAGG
Defb1	ATGGAGCCAGGTGTTGGCAT	TGGGCTTATCTGGTTTACAGGTT
Ostn	CCTGCGTCTTGATGATCTGGT	TCCATGGGAATACCAAACCGC
Gapdh	CCACCCCAGCAAGGAGAC	GAAATTGTGAGGGAGATGCT

Supplementary Table 2. Functional analysis of differentially expressed genes (DEGs) in anterior cingulate cortex (ACC), amygdala and hippocampus of the aging mice

Gene	Total	Inflammation	Apoptosis	Oxidative stress	Synaptic plasticity	Glutamate receptor	DNA methylation
Depression-related DEGs in ACC (12 M)	47	47	45	41	12	31	44
Depression-related DEGs in ACC (24 M)	96	96	85	81	36	64	88
Depression-related DEGs in amygdala (12 M)	25	25	23	20	9	18	22
Depression-related DEGs in amygdala (24 M)	103	103	90	91	42	73	94
Anxiety-related DEGs in ACC (12 M)	205	205	-	90	-	70	129
Anxiety related DEGs in ACC (24 M)	124	124	-	61	-	52	74
Anxiety-related DEGs in amygdala (12 M)	110	110	-	25	-	33	61
Anxiety-related DEGs in amygdala (24 M)	128	128	-	82	-	59	86
Cognitive dysfunction-related DEGs in hippocampus (12 M)	125	125	97	83	43	66	94
Cognitive dysfunction-related DEGs in hippocampus (24 M)	128	128	101	89	43	78	94

Supplementary Table 3. Functional analysis of the genes with differentially expressed isoforms in anterior cingulate cortex (ACC), amygdala and hippocampus of the aging mice

Gene	Total	Inflammation	Apoptosis	Oxidative stress	Synaptic plasticity	Glutamate receptor	DNA methylation
Depression-related genes in ACC (12 M)	527	527	467	419	223	354	481
Depression-related genes in ACC (24 M)	507	507	440	404	194	325	439
Depression-related genes in amygdala (12 M)	448	448	399	359	181	291	396
Depression-related genes in amygdala (24 M)	564	564	489	438	229	367	499
Anxiety-related genes in ACC (12 M)	1918	1918	-	1072	-	751	1354
Anxiety related genes in ACC (24 M)	1998	1998	-	1104	-	759	1346
Anxiety-related genes in amygdala (12 M)	1706	1706	-	992	-	646	1175
Anxiety-related genes in amygdala (24 M)	1991	1991	-	1137	-	779	1394
Cognitive dysfunction-related genes in hippocampus (12 M)	920	920	749	651	320	509	735
Cognitive dysfunction-related genes in hippocampus (24 M)	881	881	726	652	292	471	702

Supplementary Table 4. Correlation analysis between the DEGs in the anterior cingulate cortex/amygdala and behavioral phenotypes from 2- and 12-month old mice.

	Gene	Open field test			Elevated plus-maze test		Tail suspension test	Forced swimming test
		% distance in central	Entries in central	% time in central	% entries in open arms	% time in open arms	Immobile time	Immobile time
Depression related DEGs in ACC	*Esr1*						0.902 (0.014)*	0.902 (0.008)**
	Crhr2						0.927 (0.008)**	0.977 (0.001)**
	Tgif1						0.848 (0.033)*	0.912 (0.011)*
Anxiety related DEGs in ACC	*Esr1*	-0.856 (0.030)*	-0.899 (0.015)*	-0.768 (0.074)	-0.909 (0.012)*	-0.642 (0.169)		
	Crhr2	-0.716 (0.110)	-0.876 (0.022)*	-0.658 (0.155)	-0.810 (0.051)	-0.662 (0.152)		
	Tshr	-0.939 (0.006)**	-0.931 (0.007)**	-0.808 (0.052)	-0.968 (0.001)**	-0.833 (0.039)*		
Depression related DEGs in amygdala	*Vdr*						0.886 (0.019)*	0.872 (0.023)*
	Kif11						-0.850 (0.032)*	-0.823 (0.044)*
	Pomc						0.778 (0.068)	0.674 (0.142)
Anxiety related DEGs in amygdala	*Bcl2l10*	-0.858 (0.029)*	-0.884 (0.019)*	-0.879 (0.021)*	-0.852 (0.031)*	-0.647 (0.165)		
	Serpinf2	0.934 (0.006)**	0.886 (0.019)*	0.828 (0.042)*	0.852 (0.031)*	0.860 (0.028)*		
	Piwil4	0.837 (0.038)*	0.944 (0.005)**	0.890 (0.017)*	0.795 (0.059)	0.810 (0.051)		

Pearson's *r* values for the correlation are summarized and *P* values are in parentheses in the table. N = 3 biological repeats (3 mice)/age. **P* < 0.05, ***P* < 0.01.

Supplementary Table 5. Correlation analysis between the DEGs in hippocampus and behavioral phenotype from 2- and 12-month old mice.

Gene	Y-maze spontaneous alternation test	Novel-object recognition test		Morris water maze test			
	% spontaneous alternation	Recognition index (after 3 h)	Recognition index (after 24 h)	Platform crossing (after 1 h)	Platform crossing (after 24 h)	Latency to platform (after 1 h)	Latency to platform (after 24 h)
Plg	-0.892 (0.017)*	-0.956 (0.003)**	-0.854 (0.030)*	-0.889 (0.018)*	-0.726 (0.102)	0.804 (0.054)	0.746 (0.088)
Dbh	0.884 (0.019)*	0.855 (0.030)*	0.760 (0.080)	0.799 (0.057)	0.755 (0.082)	-0.672 (0.144)	-0.789 (0.062)
Prph	-0.756 (0.082)	-0.773 (0.071)	-0.784 (0.065)	-0,869 (0.025)*	-0.772 (0.072)	0.764 (0.077)	0.642 (0.101)

Pearson's *r* values for the correlation are summarized and *P* values are in parentheses in the table. N = 3 biological repeats (3 mice)/age. *P < 0.05, **P < 0.01.
